# Epidemiological and etiological aspects of burning mouth syndrome

**Published:** 2014-09-25

**Authors:** EC Coculescu, Ş Ţovaru, BI Coculescu

**Affiliations:** * Department of Oral Medicine, Faculty of Dental Medicine, “Carol Davila" University of Medicine and Pharmacy, Bucharest; ** Discipline of Microbiology, Faculty of Medicine, “Titu Maiorescu" University, Bucharest

**Keywords:** burning mouth syndrome, glossodynia, orofacial pain, neuropathic pain, etiopahogenesis

## Abstract

Abstract

Burning mouth syndrome (BMS) is defined as a chronic pain condition characterized by a burning sensation in clinically healthy oral mucosa. Incidence BMS diagnosed in the Department of Oral Medicine - Oral Pathology Dental Faculty of Medicine, "Carol Davila" University of Medicine and Pharmacy Bucharest is 16,23%. The etiology of BMS remains far less known. This article makes an overview of the latest theories about possible etiopathogenic factors involved in the occurrence of BMS.

## Introduction

First described in the nineteenth century, burning mouth syndrome (BMS) has been characterized in the early twentieth century by Butlin and Oppenheim as glossodynia, because tongue is the main headquarters of the location of pain in most patients [**1-4**]. In later years, BMS has been referred to as glossopyrosis, oral dysesthesia, sore tongue, stomatopyrosis, and stomatodynia [2,5,6].

 It is now considered a syndrome, in fact a complex of clinical symptoms [**7,8**]. BMS was first classified as a disease distinct from the International Headache Society (IHS) in 2004 [**2**].

 BMS is a chronic clinical entity manifested as burning type pain or burning sensation in the mouth without being accompanied by abnormal clinical or laboratory results [**4**].

 The International Association for the Study of Pain (IASP) has identified it as a “distinct nosological entity" characterized by “burning oral sensation or pain, unremitting while in the absence of objective clinical changes in the oral mucosa" [**4**].

 When defining BMS, the International Headache Society identifies it as “an intraoral burning sensation for which no medical or dental cause can be found" [**2**]. Therefore, BMS can be regarded as a particular form of neuropathic pain [**9**].

 Scala et al. (2003) identify as features of the syndrome – pain, located bilaterally lasting for at least 6 months of interest for oral mucosa frequently lingual, which appears to be clinically healthy [10,14]. According to Lopez-Jornet et al. (2010), BMS is considered a chronic pain disorder that affects the quality of life [**11**].

## Classification

 Depending on the etiology that it induces, BMS can be classified into two forms:

 • primary (essential / idiopathic), the organic causes local / systemic cannot be identified, but the peripheral and central neuropathic pathways are involved, and

 • secondary form determined by local factors, systemic or psychological [**14**].

 Epidemiology

 When setting epidemiology BMS is difficult and imprecise because there are no universally accepted definitions, different epidemiological studies often refer to different clinical entities where oral mucosal lesions lack. The glossodynia prevalence in the general population is estimated at 2,5 to 5,1% [**12**]. In a study by Bergdahl and Bergdahl (1999), the prevalence was estimated at 3,7% of 1427 subjects aged between 20 and 69 years [**3,12**]. This figure rises to 14% in studies by Ferguson et al. (1981) and 26% of those taken by van der Waal (1990), where the group considered consists exclusively of postmenopausal women. Hypothetically, BMS apparent association with gender, age and menopause has long been suspected because of hormonal changes that occur and which could have a pathogenic role. However, women are more affected by the disease than men, the gender ratio being 7:1. Medium age of patients was 62 years old, with a range between 40 and 85 years old [**12**].

 This condition has a high prevalence, which varies according to the studies from 0,7% to 14,8 - 15% [6,10]. Sardella et al. (2013) found a prevalence of BMS in the general population between 0,5-5% [**15**].

 The analysis of over 1000 patients randomly selected from the Swedish public dental service records revealed that a percentage of 3,7% subjects were diagnosed with BMS [**2**]. Haberland et al. (1999) found that 10% of new patients were diagnosed with BMS [**2,16**].

 More recently, a retrospective meta-study comprising over 3000 patients in Brazil refered to oral pathology service reported a prevalence of about 1% [**2**].

 Generally, the prevalence of reported BMS is between 0,7 and 5% of the population [**13**].

 Prevalence of pain syndrome in Oral Pathology Service of the Faculty of Dental Medicine, "Carol Davila" University of Medicine and Pharmacy, Bucharest is 16,23%.

 Etiology

 The etiopathogenesis of BMS is poorly known at present [**12**]. Based on the definition provided by the International Headache Society, which presumes that BMS is of idiopathic nature, attempts have been made to identify factors associated with the possible role which is etiopatologically associated to the syndrome. Acceptance of such assumptions regarding the BMS etiopathogenesis syndrome allowed the classification as primary, idiopathic and secondary forms, where the risk factors have been identified as local or systemic [**10**].

 Thus a lot of many assumptions can be made that they can be grouped into two main etiological theories [**12**]:

 1) BMS has a multifactorial origin,

 2) BMS as an expression of psychological problems.

 Those who support the multifactorial hypothesis identified a number of triggers such as local or systemic (see Table 1) which may be responsible for the sensation of burning mouth [**12**].

 a. General predisposing factors (age, sex and menopause)

 BMS has a clear predisposition related to sex and age. It can affect any age group 27-87 years old, however, the average age of reference is 61 [**2**]. BMS rarely occurs before the age of 30 [**13**]. Women are 2,5 to 7 times more frequently affected than men. In addition, up to 90% of female patients with the BMS are around menopause, with typical onset of symptoms from 3 years before and 12 after the “climacteric" [**2**].

 The explanation of BMS distribution predominantly in women during perimenopause, according to Woda et al. (2009) is that BMS is the result of hormonal changes in women during menopause, the symptoms commonly associated with chronic anxiety or stress [**17**]. Laboratory examinations revealed with the postmenopausal patients an increased salivary phosphate concentration, protein, Na+, K+, Ca2+ and Mg2+ [**18**].

 b. Possible causative factors can be subdivided into five categories (**Table 1**)

 Local factors

 Local factors have a direct effect on the oral mucosa irritation. They can be physical, chemical or biological (some bacteria or fungi).

**Table 1:**
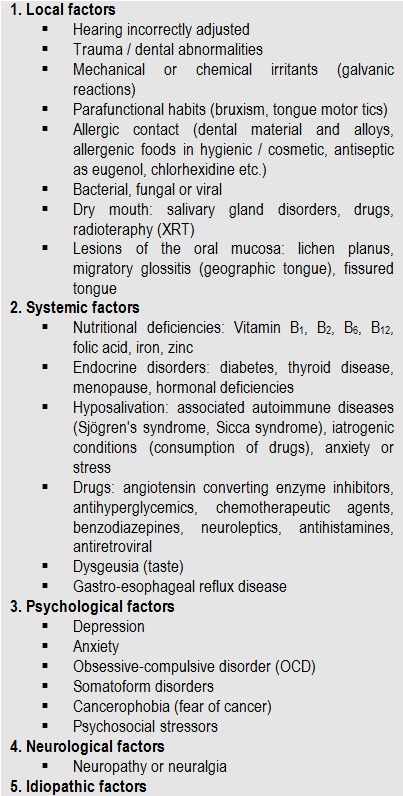
Local, systemic, psychological, neurological and idiopathic factors possible cause in BMS [**11,13,19**]

 BMS local factors involve qualitative and quantitative changes in saliva. In a study (Lamey and Lamb, 1988), 34% of 150 patients evaluated reported xerostomia or salivary protein concentration variations or electrolytes like K+, PO3- face normal [**20**]. The composition of saliva may play a role in the etiopathogenesis of BMS, some authors considering important to analyze and dose low molecular weight salivary proteins [**11,21,22**]. Other studies showed a significant increase in the level of BMS saliva Na+, lysozyme, amylase and immunoglobulins as compared to the the control group [**11**].

 Xerostomia is a common symptom associated with the BMS in patients, the prevalence ranging between 34 and 39% [**11,23,24**], while Grushka et al. (2003) found a higher percentage (at least 60% of the cases studied) [**11,25**].

 Among the local inducers of BMS there are also mechanical damages caused by the use of inappropriate dentures which can produce microtraumatisms or local erythema. They can also restrict normal activity of tongue muscles [**11**].

 Parafunctional habits of tongue or some tics (previous interposition of the tongue, bruxism, clenching of teeth etc.) were incriminated in the etiology of BMS [**11**]. These abnormal motor activities in the masticatory apparatus are chiefly associated with anxiety [**20**].

 Immunological etiology is also relevant. Food antigens as ascorbic acid, cinnamon, nicotinic acid, propylene glycol and benzoic acid as well as allergens such as dental alloys zinc, cobalt, mercury, gold, palladium, or sodium lauryl sulfate (a detergent contained in the dentifrice) are incriminated in the development of BMS [26-28]. Some authors have reported cases of healing by eliminating contact with the allergen [**11**].

 Connective tissue autoimmune diseases (Sjögren's syndrome and systemic lupus erythematosus) are also associated with BMS [**29**].

 Oral infections caused by various microorganisms have been associated with the BMS, particularly Candida albicans. Other oral infections caused by bacteria such as Enterobacter, Klebsiela, Fusobacterium and Staphylococcus aureus were found with high frequency in patients with such symptoms.

 The Helicobacter pylori, ulcer pathogenesis proscribed microorganism was isolated in biopsies of the oral mucosa and techniques of molecular biology in 86% of the patients with the BMS [**11**]. In another study, Helicobacter pylori infection was detected in 12,7% of subjects with mouth severe pain [**30**].

 In recent years, we have observed that changes in taste perception and pain tolerance could be possible causes of burning sensation. Taste is located fundamentally in the lingual fungiform papillae and severe pain in some patients with nonspecific algic symptom, especially women, there is a large number of such papillae present on the anterior part of the tongue, which is called “supertasters" [**31**].

 Systemic factors

Immunological etiology is also relevant. Food antigens like acid systemic factors involved in BMS mentioned especially vitamin deficiencies (B1, B2, B6, B12, C and folic acid) and iron deficiency (anemia). Recently, the BMS has been associated with lower serum zinc levels [**31**].

 The exact mechanism by which these nutritional deficiencies can lead to the onset of burning oral mucosa is unknown. In clinical situations characterized by sideropenic BMS in the genesis of cytochrome oxidase deficiency, a compound containing iron enzyme is incriminated. It is postulated that quantitative involvement enzyme becomes responsible for the induction of functional changes in the epithelium, and the presence of folic acid and vitamin deficits. B12 can induce changes in the morphology of the mucosa [**12**].

 Certain hormonal changes (hypoestrogenemia), diabetes mellitus, hypothyroidism and thyroid type immunological diseases affecting the endocrine glands were also described as possible causative factors of this disease [**11**].

 Regarding the relationship between diabetes and BMS various assumptions were launched, including the existence of metabolic changes in the mouth due to peripheral neuropathy, which can generate among other disturbances also a hypofunction of the salivary glands with secondary reduction of saliva [**12,33,34**].

 Research on drugs that induce syndrome BMS showed that antihypertensives are most commonly involved, mainly angiotensin converting enzyme inhibitors - ACE inhibitors - (e.g., captopril, enalapril, lisinopril), diuretics and beta blockers together. In 33% of the cases, symptoms suggestive of BMS are dose-dependent. Statistical studies have shown an association between duration of treatment and the onset of the disease [**32**].

 A recent analysis showed an increased risk of gastrointestinal and urogenital disease associated with BMS patients, the odds ratio (OR) being 3,5 and 2,9 higher than the control group [**2**].

 Psychogenic factors

 Admission psychogenic hypothesis in the pathogenesis of BMS follows from the observation that most patients with this syndrome associate mental disorders more or less obvious. Of these disorders, the literature records highlights the presence of anxiety, depression, hypochondria and oncophobia [**12**]. These psychological disorders play an important role in modulating pain perception, being able to increase or decrease nerve transmission from peripheral pain receptors and alter individual perception of pain, reduced pain threshold, so that normal stimuli should be perceived as painful [**19**].

 Almost always there are glossodynia manifestations of patients with alterations in the emotional sphere. Despite such statistical results, it was not possible to demonstrate a direct causal relationship between psychiatric disorders and BMS syndrome [**12**].

 Neuropathic basis

 The involvement of psychoneuroendocrine factor is even more difficult to explain as it has been demonstrated that BMS patients show a greater tendency to somatization and to the development of psychiatric symptoms [**11**]. For this reason, the theory that burning mouth syndrome is a form of neuropathic pain has been accepted [**15**]. Neuropathy in BMS etiopathogenesis mechanism is still controversial, the expert literature indicating the possibility of a dysfunction at the peripheral or central reflex arc path and the processing of cortical excitation [**2,35**].

 The presence of changes in taste and the fact that many patients with BMS are “supertasters" suggests an interaction between taste and nociceptive mechanisms of pain that would connect the sensory taste fibers of the chorda tympani and / or glossopharyngeal nerves to oral pain in the central nervous system. This leads to a disinhibition of the central and, therefore, hyperactivity of trigeminal nociceptive pathways which in turn produces a much more intense response to the action of irritating factors, leading to the occurrence of the oral sensation of pain [**11**].

## Conclusions

Following the evaluation of the data in the expert literature studied, it can be said that there is a lack of a universally accepted definition of BMS, and disease characteristics are still imprecisely defined. This can easily lead to misunderstandings, most of them because of the most common terms to define different clinical forms of glossodynia, they only share the burning mouth sensation. The salient features of this syndrome can be summarized in the following definition: “idiopathic pathological condition which is characterized by a burning sensation in the mouth, which appears clinically healthy" [**12**].

 When accepting such a definition, the diagnosis of primary BMS can be considered one of exclusion. The literature suggests that the diagnosis of primary BMS can be a challenge for both dentists and medical doctors, often leading to a substantial delay in the correct diagnosis and initiation of the appropriate treatment strategies [**13**].
